# Hepatic saturated fatty acid fraction is associated with de novo lipogenesis and hepatic insulin resistance

**DOI:** 10.1038/s41467-020-15684-0

**Published:** 2020-04-20

**Authors:** Kay H. M. Roumans, Lucas Lindeboom, Pandichelvam Veeraiah, Carlijn M. E. Remie, Esther Phielix, Bas Havekes, Yvonne M. H. Bruls, Martijn C. G. J. Brouwers, Marcus Ståhlman, Marjan Alssema, Harry P. F. Peters, Renée de Mutsert, Bart Staels, Marja-Riitta Taskinen, Jan Borén, Patrick Schrauwen, Vera B. Schrauwen-Hinderling

**Affiliations:** 1 Department of Nutrition and Movement Sciences, Maastricht University, P.O. BOX 616, 6200 MD, Maastricht, The Netherlands; 2 Department of Radiology and Nuclear Medicine, Maastricht University Medical Center, P.O. BOX 5800, 6202 AZ Maastricht, The Netherlands; 3 Department of Internal Medicine, Division of Endocrinology and Metabolic Disease, Maastricht University Medical Center, P.O. BOX 5800, 6202 AZ Maastricht, The Netherlands; 4 Department of Molecular and Clinical Medicine, University of Gothenburg, and Sahlgrenska University Hospital, P.O. Box 428, 40530 Gothenburg, Sweden; 5 Unilever Food Innovation Center, Plantage 14, 6708 WJ Wageningen, The Netherlands; 6 Department of Clinical Epidemiology, Leiden University Medical Center, P.O. box 9600, 2300 RC Leiden, The Netherlands; 7 Univ. Lille, Inserm, CHU Lille, Institut Pasteur de Lille, U1011—EGID, F-59000 Lille, France; 8 Research Program, Unit Clinical and Molecular Metabolism, University of Helsinki, P.O box 63 (Haartmaninkatu 8), 00014 Helsinki, Finland

**Keywords:** Type 2 diabetes, Non-alcoholic fatty liver disease

## Abstract

Hepatic steatosis is associated with poor cardiometabolic health, with de novo lipogenesis (DNL) contributing to hepatic steatosis and subsequent insulin resistance. Hepatic saturated fatty acids (SFA) may be a marker of DNL and are suggested to be most detrimental in contributing to insulin resistance. Here, we show in a cross-sectional study design (ClinicalTrials.gov ID: NCT03211299) that we are able to distinguish the fractions of hepatic SFA, mono- and polyunsaturated fatty acids in healthy and metabolically compromised volunteers using proton magnetic resonance spectroscopy (^1^H-MRS). DNL is positively associated with SFA fraction and is elevated in patients with non-alcoholic fatty liver and type 2 diabetes. Intriguingly, SFA fraction shows a strong, negative correlation with hepatic insulin sensitivity. Our results show that the hepatic lipid composition, as determined by our ^1^H-MRS methodology, is a measure of DNL and suggest that specifically the SFA fraction may hamper hepatic insulin sensitivity.

## Abstract (web summary)

Hepatic steatosis is associated with poor cardiometabolic health, with de novo lipogenesis (DNL) contributing to hepatic steatosis and subsequent insulin resistance. Here, the authors use ^1^H-MRS methodology to show hepatic SFA fraction is a measure of DNL and specifically may hamper hepatic insulin sensitivity.

## Introduction

Non-alcoholic fatty liver (NAFL) is the most common cause of chronic liver disease, with estimated prevalence rates of 20–35% in Western countries^1^. In obese people NAFL prevalence rates as high as 50–70% have been reported^2^. NAFL can progress to steatohepatitis, fibrosis, and cirrhosis, which can lead to liver failure, and hepatocellular carcinoma^3^. Moreover, ectopic fat accumulation in the liver is associated with impairments in cardiometabolic health^4,5^. In this respect, NAFL has been shown to be associated with insulin resistance on hepatic and whole-body level^6–9^.

However, not all individuals with NAFL will develop insulin resistance, steatohepatitis or other liver disease, and it is of utmost clinical importance to understand which factors contribute to a pathologic fatty liver. It has been suggested that the pathway by which fat accumulation in the liver occurs may impact the clinical outcome. It has been shown that high rates of de novo lipogenesis (DNL) are associated with metabolic risk^10–12^. In addition, animal experiments have suggested that the degree of saturation of the accumulating fatty acids in the liver may impact the metabolic consequences, with more saturated fatty acids (SFA) leading to worsened metabolic outcome^13^. Interestingly, the end product of de novo synthesis of fatty acids is mainly SFA and therefore, high rates of DNL may result in a higher proportion of hepatic SFA, possibly explaining why DNL is associated with poor metabolic health. These findings stress the importance for a more detailed characterization of hepatic lipid composition in humans, to ultimately understand the risk factors for the development of hepatic insulin resistance and disease.

However, in humans such data is very scarce, mainly due to the invasiveness of liver biopsy procedures that are needed. Therefore, only very few studies have determined hepatic fat composition, and these studies have been performed in patients in which liver biopsies were justified due to their risk for liver disease. Generally, these studies showed higher mono-unsaturated fatty acids (MUFA) fraction at the expense of the poly-unsaturated fatty acids (PUFA) fraction in people with NAFL compared to people without NAFL^14,15^. Earlier MR-based studies already investigated some parameters that are linked to degree of unsaturation^16–19^, however these do not specifically and robustly quantify hepatic SFA, MUFA, and PUFA fraction separately.

Here, we develop, validate, and apply a magnetic resonance (MR) post-processing tool that enables to non-invasively quantify the fractions of hepatic SFA, MUFA, and PUFA separately, in healthy and metabolically compromised human volunteers. Using this methodology, we test the hypothesis that higher rates of DNL are associated with an increased fraction of SFA in human liver. We also investigate if populations at higher risk to develop metabolic complications are characterized by altered hepatic fatty acid composition, and whether hepatic fatty acid composition is related to hepatic insulin sensitivity (IS). We show that our ^1^H-MRS postprocessing methodology can be used to measure hepatic fatty acid composition in humans, that hepatic SFA content is strongly related to rates of DNL, and that specifically the hepatic SFA fraction is related to hepatic insulin resistance.

## Results

### Development and validation of hepatic ^1^H-MRS method

**Fig. 1:**
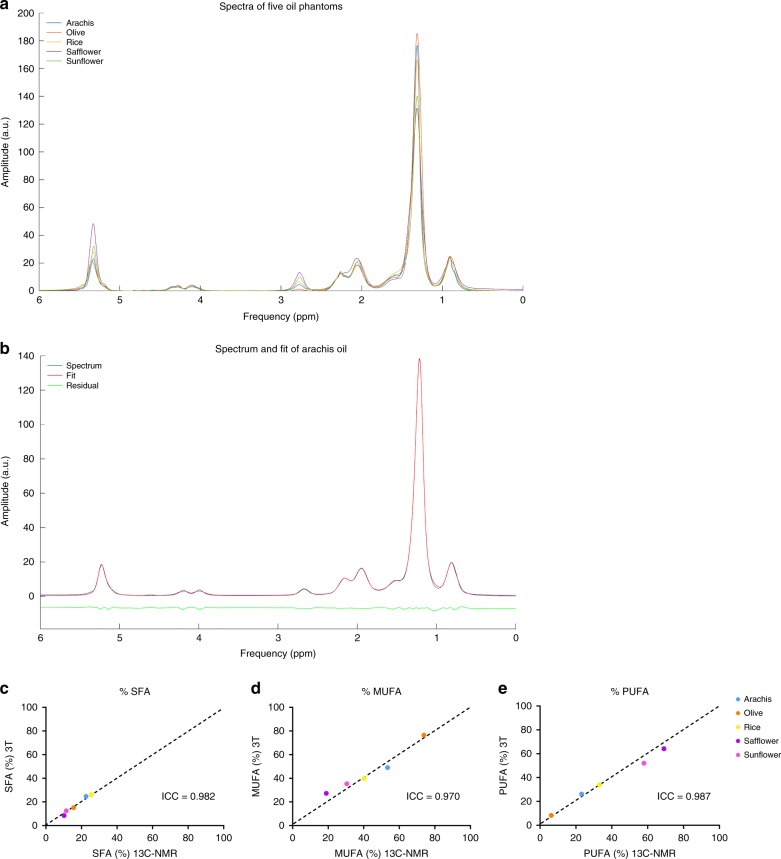
Validation of ^1^H-MRS method in oil phantoms. **a** Lipid spectra acquired from five different oil phantoms (arachis, olive, rice, safflower, and sunflower oil) showing the different lipid proton peaks and their position. **b** Lipid spectrum and fit of arachis oil. Correlations between **c** SFA, **d** MUFA, **e** PUFA measured at 3T with ^1^H-MRS and measured with high-resolution ^13^C-NMR spectroscopy. The intraclass correlation coefficient (ICC) is shown in the respective plots (Intraclass correlation).

To allow determination of hepatic fatty acid composition, we developed a ^1^H-MRS analysis protocol. To this end, we used information from ^1^H high-resolution (HR) NMR spectra from five different vegetable oils to improve our analysis routine and used ^13^C HR NMR to determine the true lipid composition of these oils. We acquired spectra from these different oils with proton magnetic resonance spectroscopy (^1^H-MRS) on our clinical 3T scanner and calculated average corrections factors to correct for TE-induced losses in the ^1^H-MRS spectra. When using the average corrections factors, the lipid composition as determined by our clinical protocol showed excellent agreement with the true lipid composition determined by HR NMR: the intraclass correlation coefficient (ICC) for SFA, MUFA, and PUFA fraction was 0.982, 0.970, 0.987, and the CV was 6%, 9%, and 9%, respectively (Fig. 1).

**Fig. 2:**
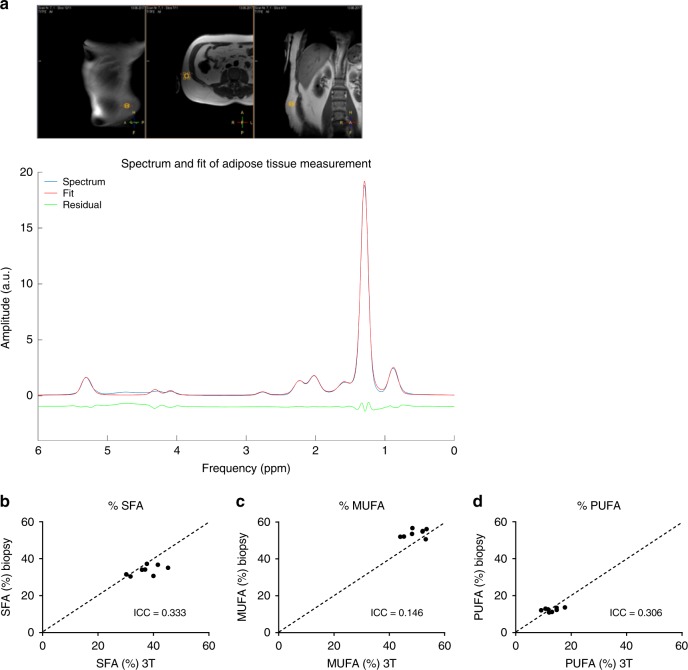
Validation of ^1^H-MRS method in subcutaneous adipose tissue. **a** T2 weighted Turbo spin echo MR image showing the voxel position located on adipose tissue and its corresponding lipid spectrum together with the fitted spectrum. The relationships between subcutaneous adipose tissue measured at 3T and adipose lipid composition determined through biopsy for the different lipid fractions: **b** SFA, **c** MUFA, and **d** PUFA (*n* = 8). The intraclass correlation coefficient (ICC) is shown in the respective plots (Intraclass correlation).

**Fig. 3:**
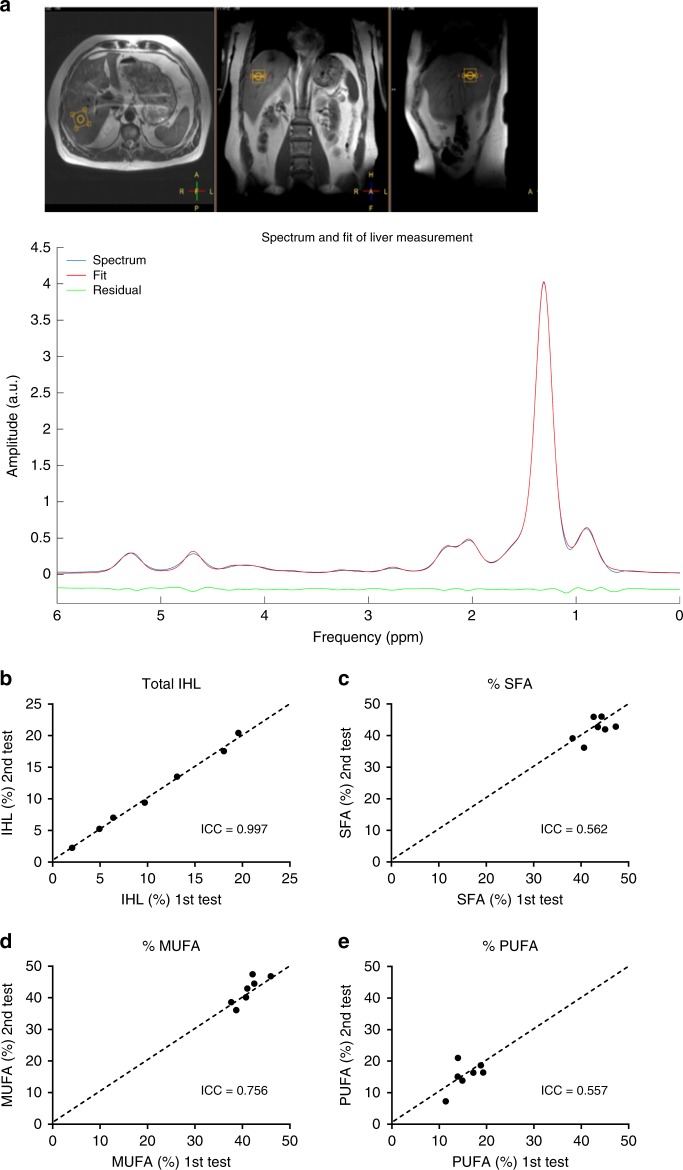
Validation of ^1^H-MRS method by testing reproducibility in vivo. **a** T2 weighted Turbo spin echo MR image showing the voxel position located on liver and its corresponding lipid spectrum together with the fitted spectrum. Scatter plots showing the reproducibility of **b** total liver fat content and **c** SFA fraction, **d** MUFA fraction, and **e** PUFA fraction (*n* = 7). Reproducibility was tested by performing two repeated measurements. The intraclass correlation coefficient (ICC) is shown in the respective plots (Intraclass correlation).

As it is known that in vivo several factors can influence MR spectra, the next step was to validate our method in vivo. To this end, we aimed to validate our ^1^H-MRS lipid composition measurement with analysis performed in biopsy material. As it is ethically difficult to take liver biopsies for this purpose, we rather performed subcutaneous adipose tissue biopsies, which is far less invasive. Therefore, we validated our MRS method in vivo by comparing lipid composition in subcutaneous adipose tissue acquired by the ^1^H-MRS technique and by mass spectrometry analysis in subcutaneous adipose tissue biopsies in eight participants (Fig. 2). As can be seen in Fig. 2, there was reasonably good agreement between the two methods. The ICC for SFA, MUFA, and PUFA fraction was 0.333, 0.146, 0.306 and the CV was 8%, 7%, 12%, respectively. Thus, lipid composition determined by ^1^H-MRS in vivo is in close agreement with lipid composition measured ex vivo in adipose tissue biopsies. The final validation step was to apply our protocol in the liver in vivo. We tested reproducibility in seven healthy individuals (separate group) with intrahepatic lipid content ranging from 2% to 18% (Fig. 3). As can be seen in Fig. 3, the ICC for total IHL content, SFA, MUFA, and PUFA fraction was found to be 0.997, 0.562, 0.756, 0.557 and the CV was 4%, 4%, 3%, and 12%, respectively. These results indicate high reproducibility for determining in vivo hepatic lipid composition using our developed approach.

**Fig. 4:**
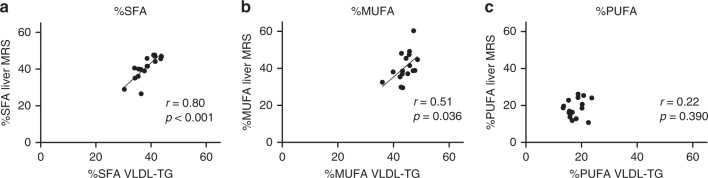
The relationship between hepatic lipid composition and plasma VLDL-TG composition. Relationships are shown for the different lipid fractions: **a** SFA, **b** MUFA, and **c** PUFA (*n* = 17). Hepatic %SFA determined with MRS and %SFA in VLDL-TG correlated significantly (*p* = 1.38 × 10^−4^). The correlation coefficient is shown in the respective plots (two-sided Pearson correlation).

In addition, we compared hepatic lipid composition by our MRS method to plasma VLDL-triglyceride (VLDL-TG) composition acquired by mass spectrometry analysis in 17 participants (Fig. 4). As can be seen in Fig. 4, especially the SFA fraction showed a strong correlation with hepatic SFA% (Pearson *r* = 0.80, *p* < 0.001).

### Higher hepatic SFA is associated with increased DNL

**Fig. 5:**
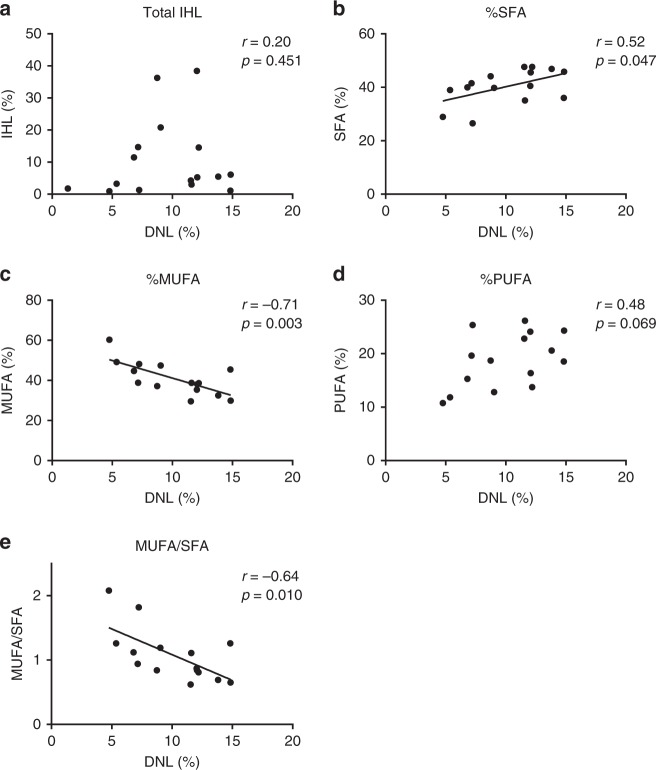
Relationship between DNL and liver fat composition. The relationships between DNL and **a** total liver fat content, **b** SFA fraction, **c** MUFA fraction, **d** PUFA fraction, and **e** MUFA/SFA ratio in healthy overweight/obese participants (with and without NAFL, *n* = 16 for total liver fat content and *n* = 15 for the fatty acid fractions and MUFA/SFA ratio). The correlation coefficient is shown in the respective plots (**a**; two-sided Spearman correlation, **b**–**e**; two-sided Pearson correlation).

DNL is an important factor in the development of fatty liver^20^. We hypothesized that DNL would specifically lead to the accumulation of SFA, as palmitate is the main product of DNL. Therefore, we determined DNL by deuterated water in overweight and obese participants with a wide range of liver fat content (*n* = 16, 0.9–38.4%) and related it to the hepatic fatty acid composition determined by our MRS protocol in the same volunteers. DNL was not associated with total liver fat content (Fig. 5a). Interestingly, however, DNL correlated positively with the hepatic SFA fraction (Pearson *r* = 0.52, *p* = 0.047; Fig. 5b). Furthermore, we found a strong negative correlation between DNL and hepatic MUFA fraction (Pearson *r* = −0.71, *p* = 0.003; Fig. 5c). In addition, DNL was negatively correlated with MUFA/SFA ratio (Pearson *r* = −0.64, *p* = 0.010; Fig. 5e). No association was found between DNL and PUFA fraction (Fig. 5d). Of note, SFA fraction in plasma VLDL-TG did not correlate with DNL (Pearson *r* = 0.27, *p* = 0.308). These results suggest that (1) higher rates of DNL lead to altered saturation of hepatic lipids and (2) that our non-invasive method can be used as a measure of the degree of DNL, directly in the liver.

### Hepatic SFA fraction is of clinical relevance in humans

To investigate if hepatic lipid composition may also have clinical relevance, we compared hepatic lipid composition in volunteers with a range in metabolic complications. We subdivided the volunteers in whom we demonstrated the relationship between DNL and hepatic SFA, into participants with NAFL (liver fat content >5%, *n* = 15) and control participants (liver fat content <5%, *n* = 7); volunteers with NAFL are known to be at increased risk to develop type 2 diabetes and other cardiometabolic disorders^6–8^. In addition, we measured hepatic lipid fractions and hepatic IS in a group of patients with type 2 diabetes (*n* = 9) and furthermore, determined hepatic lipid fractions in glycogen storage disease type 1a (GSD1a) patients (*n* = 7), known to have elevated rates of DNL. This inborn error of metabolism is caused by a defect in glucose-6-phosphatase, which hampers the final step in gluconeogenesis and glycogenolysis in liver and kidney, and, as a consequence, favors the shift of glucose-6-phosphate towards DNL and hepatic steatosis^21^. Control and NAFL participants and patients with type 2 diabetes were comparable in age and BMI. All subject characteristics are summarized in Table 1.

**Table 1:** Subject characteristics of control and NAFL participants, patients with T2D and GSD1a.

	Control (*n* = 7)	NAFL (*n* = 15)	T2D (*n* = 9)	GSD1a (*n* = 7)
Age (years)	59 ± 6.8	58 ± 7.1	65 ± 4.5	37 ± 12.3^a–c^
BMI (kg/m^2^)	29.1 ± 2.3	30.7 ± 3.1	29.4 ± 4.2	27.5 ± 3.2
Sex (f/m)	6/1	7/8	2/7	5/2
Body fat (%)	42.1 ± 8.1	42.1 ± 7.8	34.1 ± 5.8	–
Plasma glucose (mmol/L)	5.2 ± 0.4	5.6 ± 0.5	7.5 ± 1.1^a, b^	3.9 ± 0.8^a–c^
Plasma insulin (pmol/L)	35.2 ± 8.1	89.8 ± 40.4^a^	72.8 ± 58.3	13.8 ± 5.0^b, c^
Plasma NEFA (mmol/L)	667 ± 58	623 ± 141	566 ± 211	876 ± 421
Plasma TG (mmol/L)	1.5 ± 0.8	2.6 ± 1.2	1.6 ± 0.5	5.0 ± 1.5^a–c^
ALT (U/L)	22 ± 4.1	35 ± 14.4	27 ± 11.4	18 ± 4.9^b^
AST (U/L)	22 ± 3.7	28 ± 6.3	23 ± 5.9	34 ± 9.3 ^a, c^
Intrahepatic fat content (% weight/weight)	2.2 ± 1.3	14.4 ± 10.4^a^	6.9 ± 5.2	16.5 ± 18.9^a^

Data are presented as mean ± SD. Overweight/obese controls without NAFL (controls, *n* = 7), overweight/obese with NAFL (NAFL, *n* = 15), patients with type 2 diabetes (T2D, *n* = 9), and GSD type 1a (GSD1a, *n* = 7). Age was significantly lower in GSD1a compared to control (*p* = 2.5 × 10^−5^), NAFL (*p* = 3.0 × 10^−6^), and T2D (*p* = 1.47 × 10^−7^). Plasma glucose was significantly lower in GSD1a compared to control (*p* = 0.011), NAFL (*p* = 5.6 × 10^−5^) and T2D (*p* = 1.44 × 10^−10^), and was significantly higher in T2D compared to control (*p* = 4.0 × 10^−6^) and NAFL (*p* = 6.0 × 10^−6^). Plasma insulin was significantly lower in GSD1a compared to NAFL (*p* = 3.4 × 10^−5^) and T2D (*p* = 0.006), and was significantly higher in NAFL compared to control (*p* = 0.048). Plasma TG was significantly higher in GSD1a compared to control (*p* = 3.0 × 10^−6^), NAFL (*p* = 1.33 × 10^−4^), and T2D (*p* = 2.0 × 10^−6^). ALT was significantly higher in NAFL compared to GSD1a (*p* = 0.011). AST was significantly higher in GSD1a compared to control (*p* = 0.006) and T2D (*p* = 0.017). Intrahepatic fat content was significantly higher in NAFL compared to control (*p* = 0.002) and GSD1a compared to control (*p* = 0.027). Bonferroni correction was used for post-hoc analyses.

^a^Significantly different from control.

^b^Significantly different from NAFL.

^c^Significantly different from T2D (Kruskal–Wallis, *p* <  0.05 for plasma insulin, plasma NEFA, ALT and intrahepatic fat content, and one-way ANOVA, *p* < 0.05 for all other parameters).

**Fig. 6:**
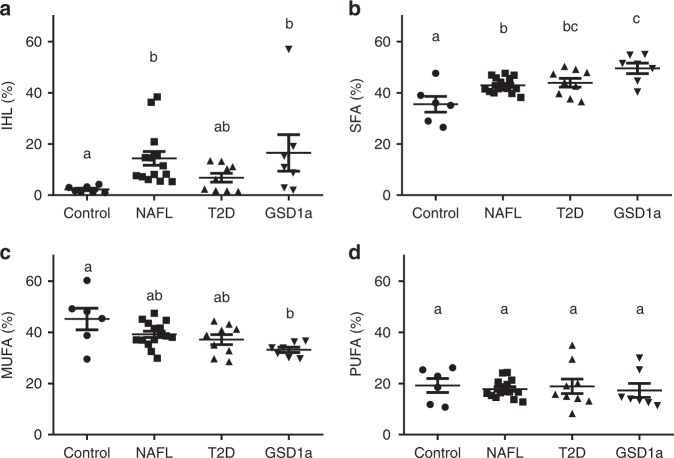
Liver fat content and composition in groups with different metabolic disorders. Comparisons between overweight/obese controls without NAFL (controls, *n* = 7 for total liver fat content, *n* = 6 for liver fat composition), overweight/obese with NAFL (NAFL, *n* = 15), patients with type 2 diabetes (T2D, *n* = 9) and GSD type 1a (GSD1a, *n* = 7). **a** Total liver fat content in control, NAFL, T2D, and GSD1a. Total liver fat content was significantly higher in the NAFL group compared to the control group (*p* = 0.002) and in the GSD1a group compared to the control group (*p* = 0.027). **b** SFA fraction in control, NAFL, T2D, and GSD1a. SFA fraction was significantly higher in the GSD1a group compared to the control group (*p* = 7.3 × 10^−5^) and NAFL group (*p* = 0.034), significantly higher in the T2D group compared to the control group (*p* = 0.016), and significantly higher in the NAFL group compared to the control group (*p* = 0.022). **c** MUFA fraction in control, NAFL, T2D, and GSD1a. MUFA fraction was significantly lower in the GSD1a group compared to the control group (*p* = 0.006). **d** PUFA fraction in control, NAFL, T2D, and GSD1a. Data are presented as mean with error bars showing the SEM. Different letters indicate significant differences between groups (Kruskal–Wallis, *p* < 0.05 for IHL and PUFA, and one-way ANOVA, *p* < 0.05 for SFA and MUFA). Bonferroni correction was used for post-hoc analyses.

Hepatic SFA fraction was higher in NAFL individuals (42.9 ± 0.8%, ANOVA, *p* = 0.022, mean ± SEM; Fig. 6b) and in type 2 diabetes patients (43.9 ± 1.7%, ANOVA, *p* = 0.016; Fig. 6b) compared to controls (35.5 ± 3.1%). Hepatic MUFA and PUFA fractions did not significantly differ between these groups (Fig. 6c and d). No significant associations were found between hepatic fat content and any of the FA fractions in healthy participants with and without NAFL. Therefore, these data suggest that specifically the SFA fraction is elevated in metabolically compromised volunteers.

To further confirm that DNL and SFA fraction may be causally related, and to further investigate the clinical relevance of SFA, we determined SFA fraction in a specific group of patients, known to have elevated rates of DNL, i.e. patients with GSD1a (*n* = 7). In these patients, SFA fraction (49.5 ± 2.0%) was markedly higher compared to NAFL participants (42.9 ± 0.8%, ANOVA, *p* = 0.034; Fig. 6b) and controls (35.5 ± 3.1%, ANOVA, *p* < 0.001; Fig. 6b). The MUFA fraction in these patients was reduced compared to controls (33.2 ± 1.1% vs. 45.2 ± 4.2%, ANOVA, *p* = 0.006; Fig. 6c). These results further confirm that the fraction of hepatic SFA and MUFA reflect the rate of DNL and can be used as a non-invasive measure to characterize hepatic metabolism in clinical patients in more detail.

### Higher hepatic SFA is associated with reduced hepatic IS

**Fig. 7:**
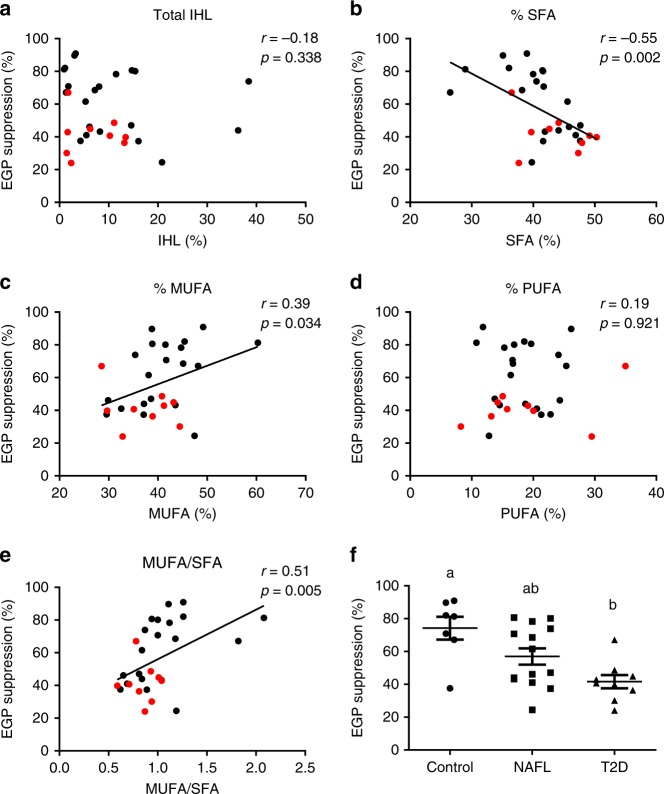
The relationship between liver fat composition and hepatic insulin sensitivity. The relationship between hepatic IS (EGP suppression) and **a** total liver fat content, **b** SFA fraction, **c** MUFA fraction, **d** PUFA fraction, and **e** MUFA/SFA ratio in overweight and obese individuals (healthy with and without NAFL in black and patients with T2D in red, *n* = 30 for total liver fat content and *n* = 29 for the fatty acid fractions and MUFA/SFA ratio). The correlation coefficient is shown in the respective plots (Two-sided Spearman correlation). **f** EGP suppression in healthy overweight/obese without NAFL (controls, *n* = 7), overweight/obese with NAFL (*n* = 14), and patients with T2D (*n* = 9). EGP suppression in patients with T2D was significantly lower compared to controls (*p* = 0.002). Data are presented as mean with error bars showing the SEM. Different letters indicate significant differences between groups (one-way ANOVA, *p* < 0.05). Bonferroni correction was used for post-hoc analyses.

Our results so far suggest that specifically the saturated fat fraction is elevated in patients and individuals with an enhanced metabolic risk. These data are consistent with findings in animal studies, where it was found that specifically hepatic SFA is of importance in relation to insulin resistance^13^. We therefore investigated the relationship between hepatic lipid composition and hepatic IS using the golden-standard two-step hyperinsulinemic euglycemic clamp. Hepatic IS was found to be 40% lower in patients with type 2 diabetes compared to controls (EGP suppression controls: 74.2 ± 7.0% and T2D: 41.6 ± 4.1%, ANOVA, *p* = 0.002; Fig. 7f), with intermediate values in NAFL (EGP suppression 56.9 ± 4.9%; Fig. 7f). The hepatic SFA fraction was strongly and negatively associated with hepatic IS (Spearman *r* = −0.55, *p* = 0.002; Fig. 7b), whereas correlation with hepatic MUFA fraction was weaker (Spearman *r* = 0.39, *p* = 0.034; Fig. 7c) and PUFA fraction and total hepatic fat content did not correlate with hepatic IS (Fig. 7a, d). Furthermore, the MUFA/SFA ratio was positively associated with hepatic IS (Spearman *r* = 0.51, *p* = 0.005; Fig. 7e). These results suggest that specifically the SFA fraction in the liver negatively contributes to hepatic IS, rather than the total amount of hepatic fat.

## Discussion

The relation of hepatic lipid composition to cardiometabolic health and specifically insulin resistance has not been investigated in humans. Due to the invasive procedures necessary and due to challenges accompanying the use of non-invasive methods, such as ^1^H-MRS, determination of hepatic lipid composition is hampered. Here, we developed, validated, and applied ^1^H-MRS methodology that enabled us to non-invasively quantify the fractions of hepatic SFA, MUFA, and PUFA separately. We showed that DNL was positively related to the hepatic SFA fraction and negatively to the MUFA fraction in overweight/obese participants, suggesting that high rates of DNL modify fatty acid composition. We confirmed this association between DNL and hepatic SFA fraction by showing increased hepatic SFA fraction in GSD1a patients, a genetic model of increased DNL. Furthermore, we showed that the hepatic SFA fraction is elevated in NAFL and type 2 diabetes patients and related to hepatic insulin resistance. These results show that our ^1^H-MRS protocol can be used to determine hepatic fat composition in humans and that particularly the SFA fraction can be seen as a non-invasive measure of DNL, directly measured in the liver. Furthermore, our results indicate that hepatic lipid composition may be a clinically important feature of hepatic fat accumulation.

Previously, a few studies have used similar approaches using ^1^H-MRS for hepatic lipid profiling, but none of these studies have reported SFA, MUFA, and PUFA fractions separately^16–19^. Even more so, in case of changes in MUFA/PUFA ratios, the calculation of the saturation index by using the methine resonance can lead to a misinterpretation of the lipid composition. We here show that by acquiring high-quality liver spectra and by applying a sophisticated post-processing method for these spectra, it is possible to differentiate between the hepatic SFA, MUFA, and PUFA fractions in vivo in subjects with and without NAFL. Implementation of this methodology in future studies makes it possible to further explore the importance of hepatic fat composition in metabolic health and the factors that could modulate fatty acid composition.

High rates of DNL may change hepatic lipid composition. The end product of the lipogenic pathway is palmitate (C16:0), which in turn can undergo elongation to stearate (C18:0) and desaturation by Stearoyl-CoA desaturase-1 (SCD1) to the MUFAs palmitoleate (C16:1 n-7) and oleate (C18:1 n-9). Previously, it has been shown that changes in DNL as determined by stable isotope techniques correlate with changes in the fraction of palmitate in VLDL-TG^22^. In line with this, DNL was positively correlated with the hepatic SFA fraction in the current study. The hepatic MUFA fraction was negatively correlated with DNL, whereas the hepatic PUFA fraction did not correlate with DNL. From these results, it follows that upon increased DNL, the production of SFA is not necessarily accompanied by subsequent desaturation to MUFAs in humans. This is in contrast to animal studies that indicate that desaturation is upregulated in parallel to upregulation of lipogenic enzymes^13^. In line, Peter et al. showed that SCD1 mRNA expression and C18:1/C18:0 ratio in TG and phospholipid fractions in human liver were not increased in individuals with NAFL^23^. Thus, handling of fatty acids originating from DNL by the liver may be different in humans and animals. Increased desaturation following lipogenesis is believed to be a rescue mechanism to reduce the negative effects exerted by SFA^13^. Further, we find higher hepatic SFA fraction in patients with NAFL and T2DM compared to controls, which is in line with earlier results, showing that the relative contribution of DNL-derived FA to VLDL-TG was around 5–10% in healthy individuals in the fasted state, whereas their contribution increased to around 20–25% in people with NAFL^11,20,24,25^. These results suggest that the negative health effects that have been attributed to increased rates of DNL may also be due to the high fraction of SFA that result from DNL. Therefore, drug development targeting DNL is promising^26–28^, although it is important to note that the evidence in the present study is only associative and future research will have to show whether decreasing the amount of SFA has beneficial effects on metabolic health. Interestingly, however, animal studies support this notion. In mice overexpressing carbohydrate-responsive element-binding protein (ChREBP), resulting in increased activity of SCD1 and higher MUFA fraction, the increased MUFA fraction at the expense of SFA fraction has been associated with increased IS, despite high amounts of total hepatic fat content^13^. Furthermore, it has been shown in vitro that specifically SFA negatively affect insulin signaling and that desaturation of fatty acids can rescue these negative effects^13^. In line with these animal and cell data, we here show that the hepatic SFA fraction was negatively correlated and the MUFA/SFA ratio positively correlated with hepatic IS in overweight and obese participants. Plasma (VLDL-)TG is often used as a surrogate for hepatic TG. In addition, with sophisticated centrifugation methods, saturation of plasma VLDL-TG can be determined. Here we showed that saturation of hepatic lipids correlated with the saturation in plasma VLDL-TG. Interestingly, however, unlike the MRS determined SFA fraction, the SFA fraction in VLDL-TG did not correlate with DNL. These results indicate, that the SFA fraction determined by MRS is a better measure of DNL compared to the SFA fraction in VLDL-TG, and thus the use of our ^1^H-MRS approach provides extra valuable information that could not be determined from plasma VLDL-TG.

In summary, we developed an MR protocol that enabled us to non-invasively quantify the fractions of hepatic SFA, MUFA, and PUFA. Applying this ^1^H-MRS methodology, we here show that the hepatic SFA fraction is positively associated with DNL, is elevated in patients with NAFL and T2D, and negatively associated with hepatic IS. These results indicate that hepatic lipid composition, as determined by our ^1^H-MRS methodology, might be used as measure of DNL and furthermore suggest that specifically the hepatic SFA fraction may determine metabolic health.

## Methods

### Clinical study design

The study was conducted at Maastricht University Medical Center, the Netherlands, between August 2017 and June 2018, and was approved by the Medical Ethical Committee of Maastricht University Medical Center. Research was performed in accordance with relevant ethical regulations regarding human research participants. The study was registered at clinicaltrials.gov with identifier NCT03211299.

### Participants

All participants recruited for this study provided written informed consent. Twenty-two healthy overweight/obese participants (BMI 27–35 kg/m^2^), aged 45–70 years with a large range in liver fat content (0.9–38.4%) were recruited for this study. Female study participants were postmenopausal. Exclusion criteria were engagement in exercise for more than 2 h per week, unstable body weight (weight loss or gain more than 3 kg in 3 months preceding enrollment), alcohol consumption more than 2 units per day, smoking more than 5 cigarettes per day, contra-indication for MRI, use of anti-coagulants, use of other medication known to interfere with the outcome parameters, diabetes, or other active disease. Participants participated in an MRS measurement, deuterated water measurement, and two-step hyperinsulinemic-euglycemic clamp, including baseline subcutaneous adipose tissue biopsy. All measurements took place within a time window of 6 weeks. Two days before each of the measurements, participants were instructed to refrain from physical exercise and alcohol consumption. The evening before the measurements, participants consumed a standardized high carbohydrate diner and fasted overnight. For group comparisons, two patient groups were included in the study: nine patients with type 2 diabetes and seven patients with GSD1a. Patients with type 2 diabetes were aged 40–75 years, had a BMI between 25 and 38 kg/m^2^ and relatively well controlled type 2 diabetes: HbA1c < 9.5%. Patients had stable dietary habits, were on treatment with oral medication only (Metformin, Tolbutamide, or Gliclazide) and did not use other medication known to interfere with the outcome parameters. Female patients were postmenopausal. Exclusion criteria were engagement in exercise for more than 3 h per week, uncontrolled hypertension, anemia, unstable body weight (weight loss or gain more than 5 kg in 3 months preceding enrollment), alcohol, or drug abuse, being vegetarian or vegan, having significant food allergies, contra-indication for MRI, use of anti-coagulants and clinically relevant active disease. GSD1a patients were recruited aged 18 years and older, clinically diagnosed with GSD1a and without contra-indication for MRI. In vivo validation in adipose tissue was performed in a subgroup of the healthy overweight/obese participants (*n* = 8). Reproducibility measurements in the liver were performed in a separate group of individuals aged between 25–71 years and BMI between 25.7–38.5 kg/m^2^ (*n* = 7).

### Overview of specified outcomes

The primary outcome was lipid composition (SFA fraction) as measured by MRS and rates of DNL as measured by incorporation of deuterated water. Secondary outcome was hepatic IS as determined by suppression of hepatic glucose output during the low insulin phase in a two-step hyperinsulinemic-euglycemic clamp.

### Measurement of lipid content and lipid composition

In this study, the lipid content and lipid composition (fraction of hepatic SFA, MUFA, and PUFA) were determined by proton magnetic resonance spectroscopy (^1^H-MRS) (healthy overweight/obese controls without NAFL: *n* = 7 for total liver fat content, *n* = 6 for liver fat composition, healthy overweight/obese with NAFL: *n* = 15, patients with type 2 diabetes: *n* = 9 and patients with GSD type 1a: *n* = 7). All ^1^H-MRS experiments were performed on a 3T MR system (Achieva 3T-X Philips Healthcare, Best, Netherlands) by using a 32-channel sense cardiac/torso coil (Philips Healthcare, Best, Netherlands). All spectra were obtained by using a STEAM sequence^29^ with the following parameters; repetition time (TR) 4500 ms/echo time (TE) 20 ms/mixing time (TM) 16 ms, spectral bandwidth 2000 Hz and data points 2048. For the in vivo hepatic lipid spectra VAPOR water suppression^30^ was applied and an additional water reference scan was obtained. The number of averages was 16 for the phantom experiments and in adipose tissue and 128 for the in vivo hepatic spectra. We used a voxel size of 30 × 30 × 30 mm for the hepatic and 15 × 15 × 15 mm for the adipose tissue measurements.

All obtained lipid spectra were post-processed in a home-written MATLAB (MATLAB 2014b, The MathWorks, Inc., Natick, MA, USA) script in which, prior to fitting, all spectra are individually corrected for phase and frequency shift. Additionally, we performed eddy current correction for the individual lipid spectra. Phasing, frequency alignment, and eddy current correction were all performed on individual spectra before signal averaging. Bad quality spectra from in vivo (e.g. due to motion) were removed automatically. For this, the linewidth, amplitude, and frequency offset of the peaks in each individual spectrum were compared to the average values of all spectra.

For the setup of the post-processing routine we used a four-step approach. First, we acquired both ^1^H and ^13^C HR NMR spectra from five different oils (olive, arachis, sunflower, safflower, and rice oil), by using a pulse-acquire sequence. The lipid composition of the different oils was determined by integration of the peaks in the methyl region in the ^13^C HR spectra. In the ^1^H HR spectra four different regions were integrated, corresponding to the methyl protons (around 0.90 ppm), the allylic protons (around 2.02 ppm), the alpha carbonyl group (around 2.20 ppm), and the diallylic protons (around 2.75 ppm) (Supplementary Fig. 1). The ^1^H HR spectra were used to develop a basis set used by the developed fitting algorithm. The lipid signal was described by 16 individual resonances (2 for the methyl group, 5 for the methylene group, 1 for the beta methyle-group, 2 for the allylic group, 2 for the alpha-carbonyl group, and 1 for the diallylic group). In this basis set, the relative frequency shifts, the splitting patterns and the initial linewidths were described.

In a second setup step, we acquired spectra from the different oils on our clinical 3T scanner. Eddy current correction was applied based on an additional water reference scan with an identical experimental setup. The oil spectra were fitted with the developed Matlab algorithm. To this end, using the basis set from the HR spectra, the time domain signal was simulated. The amplitudes and relative frequency shifts of the individual peaks were updated iteratively. In each step the difference between the simulated spectrum and the acquired spectrum was minimized in the frequency domain. Next to these individual parameters, also the Gaussian line broadening, the Lorentzian line broadening, the zero order phase, an overall frequency shift and the baseline offset were automatically updated, affecting all the peaks in an identical fashion. Essential for our approach, we furthermore fixed the ratio of the methyl group and the alpha-carbonyl group (which overlaps with the allylic group at 3T) to 0.6. Theoretically this factor should be 0.67, as the methyl group contains three protons and the alpha-carbonyl group contains two protons and both groups are present only once in every fatty acid. However, due to TE-induced losses this factor will be affected. Therefore, this factor was determined empirically. In a final fine-tuning step of the fitting routine, the zero-order phase was accurately fitted by minimizing the residual in the spectrum in the methyl and allylic + alpha-carboxyl region specifically.

The signal amplitude ratio of the diallylic over the methyl peaks and the ratio of the alpha-carbonyl + allylic over the methyl peaks was determined in both the phantom and the HR experiment, to empirically determine correction factors for TE-induced signal in our STEAM recordings. These empirically determined correction factors were then used for the measurements in adipose tissue and in the liver to calculate the lipid composition using the following formulas:

$${\mathrm{\% }}\,{\mathrm{{PUFA}}} = {\mathrm{{CA}}} \ast \left( {\frac{2}{3} \ast \frac{{\mathrm{{{Sdiallylic}}}}}{{\mathrm{{{Smethyl}}}}}} \right) \ast 100$$

$${\mathrm{\% }}\,{\mathrm{{MUFA}}} = {\mathrm{{CB}}} \ast \left[ {\frac{3}{4} \ast \left( {\frac{{{\mathrm{{Salphacarb}}} + {\mathrm{{Sallylic}}}}}{{{\mathrm{{Smethyl}}}}} - \frac{2}{3}} \right) \ast 100} \right] - {\mathrm{\% }}{\mathrm{{PUFA}}}$$

$${\mathrm{\% }}\,{\mathrm{{SFA}}} = 100 - {\mathrm{\% }}\,{\mathrm{{PUFA}}} - {\mathrm{\% }}\,{\mathrm{{MUFA}}}$$

with CA and CB the empirically determined correction factor for the PUFA and MUFA calculation, respectively (CA = 0.83 and CB = 0.99; see also Supplementary Table 1).

In a third step, the lipid composition in subcutaneous adipose tissue was determined in vivo with ^1^H-MRS using the developed fitting routine and this was compared to the ex vivo lipid composition as determined by mass spectrometry analysis in adipose tissue biopsies, in eight participants. For the adipose tissue ^1^H-MRS measurement, we applied a gradient cycled STEAM sequence, to correct for eddy currents, as previously described^31^.

In a final validation step to determine reproducibility, we applied the developed methodology in the liver of seven healthy individuals (BMI 30.6 ± 3.7 kg/m^2^; age 49 ± 17.2 year; 3 women), with a wide range of intrahepatic fat (2.1–19.6%). To this end, we performed the ^1^H-MRS acquisition and repeated this after replacement of the subject on the table. The ICC was calculated for the lipid composition and total lipid content as a measure to determine the reproducibility between two consecutive measurements. Lipid content was calculated after T_2_ correction as ratio of the CH_3_ peak relative to the unsuppressed water resonance, expressed as percentage weight/weight.

### Deuterated water measurement of DNL

A background blood sample was drawn in the afternoon before intake of the deuterated water in 18 healthy overweight/obese participants. Together with the evening meal, participants were given 2.86 g/kg body weight deuterated water (70% 2H_2_O, Cambridge Isotope laboratories) in two servings. A blood sample for DNL analysis was drawn at fasting in the morning, 16 h after the first serving of deuterated water. The DNL was analyzed from enrichment in VLDL-TG of deuterated water^10,32^. Two participants were excluded from the analysis of DNL because of TG levels higher than 4 mmol/L, influencing the reliability of the deuterated water measurement.

### Body composition

Body mass and body volume were assessed using air-displacement plethysmography (ADP) using the Bod Pod device (Cosmed, Italy, Rome) according to the manufacturers instructions on the day of the hyperinsulinemic-euglycemic clamp (healthy overweight/obese volunteers: *n* = 21, patients with type 2 diabetes: *n* = 9)^33^. Thoracic gas volume was predicted based on equations included in the Bod Pod software (version 4.2.0). From these data, body fat percentage was calculated as described by Siri^34^.

### Subcutaneous adipose tissue biopsy

In a subgroup of eight healthy overweight/obese participants, a subcutaneous adipose tissue biopsy (~1 g) was collected 6–8 cm lateral from the umbilicus, under local anesthesia (1% lidocaine) by needle biopsy before the start of the hyperinsulinemic-euglycemic clamp. Upon tissue collection, the tissue was rinsed with sterile saline and visible blood vessels were removed. Thereafter, the biopsy was snap-frozen in liquid nitrogen and stored at 80 °C for later lipid composition analyses.

### Analysis of VLDL and adipose tissue triglycerides

Plasma samples used for VLDL-TG analyses were taken around 16 pm before the intake of the deuterated water. The VLDL fractions (20–50 µl) and adipose tissues (10–20 mg) were extracted using the BUME method^35,36^. The total lipid extracts were evaporated under a stream of nitrogen and reconstituted in 250 µL chloroform/methanol [2:1]. Triglycerides were detected by direct infusion (shotgun) analysis on a QTRAP 5500 mass spectrometer (Sciex, Concord, Canada) equipped with a robotic nanoflow ion source, TriVersa NanoMate (Advion BioSciences, Ithaca, NJ) according to previous work^37^. More specifically, a fraction of the total lipid extract was diluted 1:100 (for VLDL) and 1:100,000 (for adipose tissue) in chloroform:methanol [1:2] with 5 mM ammonium acetate and infused at 250 nl/min for 15 min. The NanoMate was run with a voltage of 1.2 kV and a gas pressure of 0.8 psi. The analysis was performed in positive ion mode by neutral loss detection of 11 common acyl fragments formed during collision-induced dissociation of the ammoniated triglycerides. The nano-interface of the mass spectrometer was heated to 60 °C and the measurements was made using a scan speed of 200 Da/s. The neutral loss scans were cycled and in total 50 cycles were acquired for each neutral loss scan (one neutral loss for each fatty acid). The data was processed using the LipidView 1.2 software (Sciex, Concord, Canada) and the most abundant signal intensities from the most commonly found triglyceride species were used to calculate the abundance and composition of the different triglycerides (Supplementary Tables 2 and 3). Quantification was made using a one point calibration against glyceryl-d_5_-hexadecanoate (CDN isotopes, Quebec, Canada), which was added to the infusion solvent.

### Hyperinsulinemic-euglycemic clamp

A two-step hyperinsulinemic-euglycemic clamp was performed to assess hepatic IS using the low-dose phase (10 mU/m^2^/min) (healthy overweight/obese participants: *n* = 21). A primed continuous infusion of D-[6,6-2 H2]glucose (0.04 mg/kg/min) was started to determine rates of endogenous glucose production (EGP), glucose appearance (Ra), and glucose disposal (Rd). After 180 min, participants were given infusion of low insulin (10 mU/m^2^ /min) for 3 h. During the last 30 min of the insulin infusion step, blood samples were collected. Steele’s single pool non-steady-state equations were used to calculate glucose Ra and Rd^38^. Volume of distribution was assumed to be 0.160 L/kg for glucose. Hepatic IS was calculated as the percentage of EGP suppression during the low-dose phase. In addition, hepatic IS was determined according to this methodology in patients with type 2 diabetes (*n* = 9). Clamp parameters are shown in Supplementary Table 4.

### Statistical analysis

Results are expressed as mean ± SEM for group comparisons. Population characteristics are expressed as mean ± SD. Continuous variables were tested for normality. Two-sided Pearson correlation was performed to identify correlations between variables. For non-normally distributed data two-sided Spearman correlation was performed. Group comparisons were assessed by one-way ANOVA. For non-normally distributed data Kruskal–Wallis analyses was performed to compare groups. In case of significant group differences in the group comparisons, post-hoc analyses were performed using Bonferroni correction to test which groups were significantly different. A *p*-value < 0.05 was considered statistically significant. Statistical analyses were performed using SPSS 23.0 for Mac OS.

## Supplementary information

Supplementary Information

## Data Availability

The source data underlying Figs. 1–7, Table 1 and Supplementary Table 4 are provided as a Source Data file and are available from the corresponding author upon reasonable request.

## Code availability

Insights in the details of the code is available upon request.
