# Supplementary material for: Hepatic saturated fatty acid fraction is associated with de novo lipogenesis and hepatic insulin resistance
Source: Nat Commun. 2020 Apr 20;11:1891. doi: 10.1038/s41467-020-15684-0 (PMC7170906; doi:10.1038/s41467-020-15684-0)
Supplement: Supplementary file 1 — Supplementary Information [file 41467_2020_15684_MOESM1_ESM.pdf]

Hepatic saturated fatty acid fraction is associated with de novo lipogenesis and hepatic insulin resistance. Roumans et al.

Supplementary information

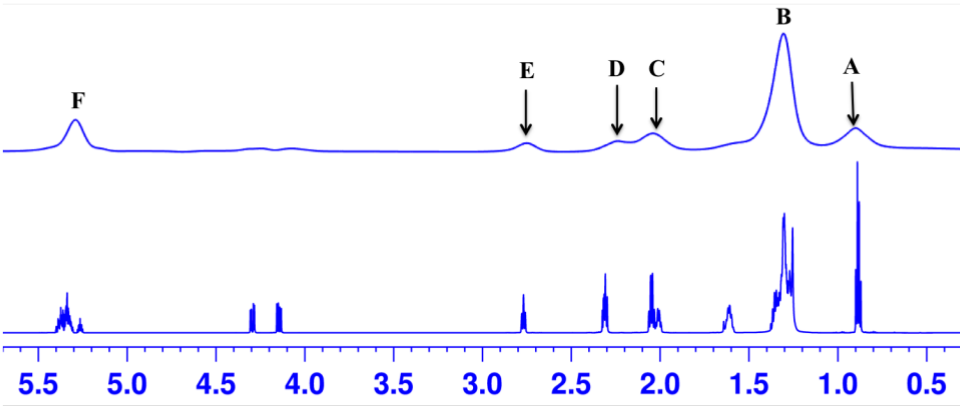

Supplementary Figure 1: Comparison of lipid spectrum acquired at 3T and high-resolution ^{1}H-NMR. Lipid spectrum acquired from the sunflower oil at 3T (top) and high-resolution ^{1}H-NMR spectrum (bottom) showing the variation in spectra resolution. A-methyl (-CH_3); B-methylene (-CH_2); C-allylic (CH_3-CH=CH-CH_2); D-alpha carbonyl (CH_2-COO); E-diallylic (=CH-CH_2-CH=); F-methine (CH=CH).

Supplementary Table 1: Correction factors for PUFA and MUFA calculation

	CA	CB
Olive oil	0,759	0,997
Arachis oil	0,735	0,987
Sunflower oil	0,907	1,003
Safflower oil	0,889	0,982
Rice oil	0,849	0,990

Supplementary Table 2: List of triglyceride species determined by mass spectrometry

Triglyceride species
TAG 48:0
TAG 48:1
TAG 48:2
TAG 48:3
TAG 50:0
TAG 50:1
TAG 50:2
TAG 50:3
TAG 50:4
TAG 50:5
TAG 52:0
TAG 52:1
TAG 52:2
TAG 52:3
TAG 52:4
TAG 52:5
TAG 52:6
TAG 54:1
TAG 54:2
TAG 54:3
TAG 54:4
TAG 54:5
TAG 54:6
TAG 54:7
TAG 56:2
TAG 56:3
TAG 56:4
TAG 56:5
TAG 56:6
TAG 56:7
TAG 56:8
TAG 58:7
TAG 58:8
TAG 58:9

Supplementary Table 3: List of fatty acid species determined by mass spectrometry

FA species	Saturation
FA 14:0	SFA
FA 16:0	SFA
FA 18:0	SFA
FA 16:1	MUFA
FA 18:1	MUFA
FA 18:2	PUFA
FA 20:3	PUFA
FA 20:4	PUFA
FA 20:5	PUFA
FA 22:5	PUFA
FA 22:6	PUFA

Supplementary Table 4: Clamp parameters in control participants, and patients with NAFL and T2D.

	Control (n=7)	NAFL (n=14)	T2D (n=9)
Ra (µmol/kg/min)			
Baseline	9.74 ± 2.40	7.64 ± 1.50	12.84 ± 2.03 [A,B]
Low insulin	11.88 ± 1.34	9.08 ± 1.69[A]	9.83 ± 2.44
High insulin	35.16 ± 11.29	26.58 ± 7.74	23.02 ± 10.76
Rd (µmol/kg/min)			
Baseline	8.87 ± 2.06	7.18 ± 1.64	13.28 ± 3.10 [A,B]
Low insulin	12.68 ± 1.30	9.04 ± 1.58 [A]	10.28 ± 2.47 [A]
High insulin	35.41 ± 10.80	26.16 ± 7.51	23.55 ± 9.93 [A]
EGP (µmol/kg/min)			
Baseline	9.74 ± 2.40	7.64 ± 1.50	12.84 ± 2.03 [A, B]
Low insulin	2.68 ± 2.55	3.15 ± 1.22	7.54 ± 2.09 [A, B]
High insulin	0.98 ± 1.47	1.07 ± 0.81	2.73 ±0.51 [A, B]
% suppression low insulin	74.20 ± 18.40	56.92 ± 18.50	41.61 ± 12.15 [A]
% suppression high insulin	89.33 ± 15.82	85.24 ± 12.06	78.44 ± 4.03

Data are presented as mean ± SD. Overweight/obese controls without NAFL (controls, n=7), overweight/obese with NAFL (NAFL, n=14), patients with type 2 diabetes (T2D, n=9). Ra baseline was significantly higher in T2D compared to control (p=0.010) and NAFL ($p=2.0x10^{-6}$). Ra low insulin was significantly lower in NAFL compared to control (p=0.010). Rd baseline was significantly higher in T2D compared to control (p=0.002) and NAFL ($p=3.0x10^{-6}$). Rd low insulin was significantly higher in control compared to NAFL (p=0.001) and T2D (p=0.045). Rd high insulin was significantly lower in T2D compared to control (p=0.045). EGP baseline was significantly higher in T2D compared to control (p=0.010) and NAFL ($p=2.0x10^{-6}$). EGP low insulin was significantly higher in T2D compared to control ($p=2.35x10^{-4}$) and NAFL (p=0.001). EGP high insulin was significantly higher in T2D compared to control (p=0.003) and NAFL (p=0.001). EGP suppression low insulin was significantly lower in T2D compared to control (p=0.002). [A] Significantly different from control, [B] significantly different from NAFL (Kruskal-Wallis p<0.05 for EGP Low Insulin and EGP suppression high insulin, and one-way ANOVA p<0.05 for all other parameters). Bonferroni correction was used for post-hoc analyses.
